# Combined therapy with adipose tissue-derived mesenchymal stromal cells and meglumine antimoniate controls lesion development and parasite load in murine cutaneous leishmaniasis caused by *Leishmania amazonensis*

**DOI:** 10.1186/s13287-020-01889-z

**Published:** 2020-08-31

**Authors:** Tadeu Diniz Ramos, Johnatas Dutra Silva, Alessandra Marcia da Fonseca-Martins, Juliana Elena da Silveira Pratti, Luan Firmino-Cruz, Diogo Maciel-Oliveira, Julio Souza Dos-Santos, João Ivo Nunes Tenorio, Almair Ferreira de Araujo, Célio Geraldo Freire-de-Lima, Bruno Lourenço Diaz, Fernanda Ferreira Cruz, Patricia Rieken Macedo Rocco, Herbert Leonel de Matos Guedes

**Affiliations:** 1 Grupo de Imunologia e Vacinologia, Laboratório de Imunofarmacologia, Instituto de Biofísica Carlos Chagas Filho, Universidade Federal do Rio de Janeiro (UFRJ), Rio de Janeiro, Brazil; 2 Laboratório de Imunomodulação, Instituto de Biofísica Carlos Chagas Filho, Universidade Federal do Rio de Janeiro (UFRJ), Rio de Janeiro, Brazil; 3 Laboratório de Investigação Pulmonar, Instituto de Biofísica Carlos Chagas Filho, Universidade Federal do Rio de Janeiro (UFRJ), Rio de Janeiro, Brazil; 4 Laboratório de Inflamação, Instituto de Biofísica Carlos Chagas Filho, Universidade Federal do Rio de Janeiro (UFRJ), Rio de Janeiro, Brazil; 5 National Institute of Science and Technology for Regenerative Medicine, Rio de Janeiro, Rio de Janeiro Brazil; 6 UFRJ Campus Duque de Caxias Professor Geraldo Cidade, Duque de Caxias, Rio de Janeiro, Brazil; 7 Laboratório Interdisciplinar de Pesquisas Médicas, Instituto Oswaldo Cruz/FIOCRUZ, Rio de Janeiro, Brazil

**Keywords:** Leishmaniasis, Mesenchymal stromal cells, Adipose tissue-derived mesenchymal stromal cells, Meglumine antimoniate, Bone marrow-derived mesenchymal stromal cells, Parasite load

## Abstract

**Background:**

Leishmaniasis is a neglected disease caused by *Leishmania* spp. One of its characteristics is an imbalance of host immune responses to foster parasite survival. In this setting, mesenchymal stromal cells (MSCs) may be a viable therapeutic alternative, given their well-established immunomodulatory potential. In this study, we compared the effects of therapy with bone marrow (BM)- and adipose tissue (AD)-derived MSCs in leishmaniasis caused by *Leishmania amazonensis* in C57BL/6 mice. After determining the most effective MSC source, we then combined these cells with meglumine antimoniate (a pentavalent antimonial commonly used for the treatment of leishmaniasis) to treat the infected mice.

**Methods:**

In vitro, co-culture of AD-MSCs and BM-MSCs with *Leishmania amazonensis-*infected macrophages was performed to understand the influence of both MSC sources in infected cells. In vivo, infected C57BL/6 mice were treated with phosphate-buffered saline (PBS), AD-MSCs and BM-MSCs, and then meglumine antimoniate was combined with MSCs from the most effective source.

**Results:**

In vitro, co-culture of *Leishmania amazonensis*-infected macrophages with BM-MSCs, compared to AD-MSCs, led to a higher parasite load and lower production of nitric oxide. Fibroblasts grown in conditioned medium from co-cultures with AD-MSCs promoted faster wound healing. Despite a non-significant difference in the production of vascular endothelial growth factor, we observed higher production of tumor necrosis factor-α and interleukin (IL)-10 in the co-culture with AD-MSCs. In vivo, treatment of infected mice with BM-MSCs did not lead to disease control; however, the use of AD-MSCs was associated with partial control of lesion development, without significant differences in the parasite load. AD-MSCs combined with meglumine antimoniate reduced lesion size and parasite load when compared to PBS and AD-MSC groups. At the infection site, we detected a small production of IL-10, but we were unable to detect production of either IL-4 or interferon-γ, indicating resolution of infection without effect on the percentage of regulatory T cells.

**Conclusion:**

Combination treatment of cutaneous leishmaniasis with AD-MSCs and meglumine antimoniate may be a viable alternative.

## Background

Leishmaniasis is one of the most important neglected tropical diseases, ranking second in the number of deaths among parasitic diseases, surpassed only by malaria [1]. In Brazil, *Leishmania* (*Leishmania*) *amazonensis* is one of the main etiological agents of leishmaniasis. This species is part of the *Leishmania mexicana* complex and is an etiological agent for a broad spectrum of leishmaniasis pathologies, including diffuse cutaneous leishmaniasis [2, 3].

The first choice for the treatment of leishmaniasis is meglumine antimoniate, a pentavalent antimonial. Although these drugs were developed in the late 1940s, they still form the cornerstone of treatment for leishmaniasis worldwide [4]. These drugs inhibit the synthesis of ATP and GTP and interfere with the bioenergetics and the redox balance of the amastigote form of the parasite [5]. However, there are several issues associated with this treatment, such as daily parenteral dosing over a long period of time and high toxicity [6]. Alternative drugs for leishmaniasis treatment, such as amphotericin B and pentamidines, present similar problems [4, 6, 7]. Therefore, the search for new therapeutic strategies with fewer side effects remains an area of active interest.

Mesenchymal stromal cells (MSCs) can be obtained from different tissues, such as the bone marrow, dental tissue, adipose tissue, and umbilical cord. These cells have been increasingly studied as a promising strategy for the treatment of various diseases [8–11], including parasitic diseases such as malaria [12] and Chagas disease [13–16].

*Leishmania amazonensis* uses mechanisms which subvert immune function in its favor to escape, survive, and establish infection [17–19]. MSCs have been described as cells with high immunomodulatory potential, regulating many factors and cell responses in different diseases [20–22]. One of these functions is the stimulation of regulatory T cell (T_reg_, CD4^+^ CD25^+^ FoxP3^+^) proliferation [23–25]. T_regs_ have been shown to play an essential role in lesion resolution in the C57BL/6 mouse model of *Leishmania amazonensis* infection [26].

The immunomodulatory potential of bone marrow-derived mesenchymal stromal cells (BM-MSCs) was first tested in vitro using co-culture with *Leishmania amazonensis*-infected peritoneal macrophages. We observed increased intracellular amastigote counts in macrophages co-cultured with BM-MSC [27]. We then evaluated BM-MSCs as a treatment in the BALB/c mouse model of *Leishmania amazonensis* infection [27], a well-established model for this parasite species [28, 29]. Although there was an increase in the number of T_regs_ upon treatment, this was insufficient to control the parasite load in this mouse model [27]. Still, it should be noted that, unlike in C57BL/6 mice, the role of T_regs_ in lesion resolution in infected BALB/c mice is still unclear. The increase in T_regs_ observed in the BALB/c model suggested that treatment with MSCs in the C57BL/6 model could serve a greater purpose in the control of *Leishmania amazonensis* infection. Furthermore, the immunomodulatory properties of MSCs can vary according to their source [30–32]. Therefore, we hypothesized that MSCs derived from adipose tissue (AD-MSCs) or bone marrow (BM-MSCs) might exert different effects in a murine model of leishmaniasis.

The objective of our study was to evaluate the effects of bone marrow-derived mesenchymal stromal cells or adipose tissue-derived mesenchymal stromal cells in C57BL/6 mice infected with *Leishmania amazonensis*. For this purpose, we evaluated the in vitro influence of BM-MSCs versus AD-MSCs on *Leishmania amazonensis*-infected macrophages. Subsequently, the therapeutic effects of MSCs from both sources were analyzed in infected C57BL/6 mice. Finally, meglumine antimoniate was combined with MSCs from the most effective source and tested in these mice.

## Methods

### Animals

To carry out the experiments in this work, we used isogenic mice of the C57BL/6 strain, originally obtained from the Animal Laboratory of Universidade Federal Fluminense (UFF) at age 4 to 6 weeks. They were maintained in our own facilities under special temperature conditions (23–25 °C), a 12/12-h light/dark cycle, and access to water and food ad libitum. At age 6 to 8 weeks, animals were used for experiments. All experimental protocols used in this work were approved by the Ethical Committees for Experimental Animal Use of Instituto de Biofísica Carlos Chagas Filho (CEUA IBCCF, protocol 157) and the Federal University of Rio de Janeiro Center of Health Sciences (CEUA-UFRJ, protocol 110/17).

### Parasites

The parasites used in the experiments presented in this paper were *Leishmania amazonensis* (MHOM/BR/75/Josefa strain) obtained by puncture of amastigotes from lesions of infected BALB/c mice. The promastigotes were maintained at 26 °C in M199 medium (Difco) containing 10% fetal bovine serum (FBS; Cultilab) until the fifth passage in culture to perform infection experiments. Infections were carried out with stationary phase culture, between the fourth and fifth days of culture.

### Preparation of MSCs

For extraction of mesenchymal cells, male C57BL/6 mice (weight 20–25 g, age 8 weeks) were used as donors. The animals were anesthetized with intravenous ketamine (25 mg/kg; Sigma) and xylazine (2 mg/kg, Sigma) and euthanized with sevoflurane (Sevorane®, Abbott). With the aid of sterile forceps and scissors, an abdominal cut was made to remove the testicles. The fat located around the animals’ epididymis was extracted and suspended in phosphate buffer (PBS). Then, the skin and muscles adjacent to the tibia and femur were removed.

To obtain AD-MSCs, the epididymal fat pads were collected, rinsed with PBS, transferred to a Petri dish, and cut into small pieces (approximately 0.2–0.8 cm^2^). The dissected pieces were washed with PBS, cut into smaller fragments, and subsequently digested with type I collagenase (1 mg/mL in DMEM/10 mM HEPES) for 30–40 min at 37 °C. Any gross remnants that persisted after collagenase digestion were poured off between 1 and 3 min, and the supernatant was transferred to a new tube containing fresh medium and centrifuged at 400×*g* for 10 min at 25 °C. The pellets were resuspended in 3.5 mL Dulbecco’s modified Eagle’s medium (DMEM; Invitrogen) containing 1% penicillin and streptomycin, with concentrations of 5000 IU/mL and 5000 μg/mL, respectively (Gibco), 20% of inactivated FBS (Invitrogen), and 15 mM HEPES (Sigma), seeded in T25 flasks (4 mL per flask), and incubated at 37 °C in a humidified atmosphere containing 5% CO_2_. On day 3 of culture, the medium was changed, and non-adherent cells were removed. Adherent cells exhibited similar proliferation rates and, upon reaching 80% confluence, they were passaged with a 0.25% trypsin-EDTA solution (Gibco) and maintained in DMEM with 10% FBS (complete medium).

For the isolation of BM-MSCs, the epiphyses of the tibia and femur were cut, and the bones placed individually in sterile tips inside sterile, 15-mL conical polystyrene tubes (TPP). The tubes were centrifuged at 1200 rpm for 5 min at room temperature. After centrifugation, the tips containing the bones were removed, and the pellets formed were suspended in low-glucose DMEM culture medium (Dulbecco’s modified Eagle medium; Invitrogen) containing 20% fetal bovine serum (Invitrogen) and 1% antibiotics (streptomycin and penicillin) (Gibco). The cell suspension was then plated in a 25-cm^2^ plastic culture bottle (TPP) and incubated at 37 °C in a humidified atmosphere containing 5% CO2.

Approximately 1 × 10^6^ cells were characterized as MSCs at the third passage according to the consensus of the International Society of Cell Therapy [33].

### Characterization of MSCs

Mesenchymal stromal cells were characterized by their ability to differentiate into osteocytes, chondrocytes, and adipocytes [33, 34]. Osteogenic differentiation was induced by culturing MSCs for up to 3 weeks in DMEM 10% FBS and 15 mM HEPES (Sigma), supplemented with 10–5 mM/L dexamethasone (Sigma), 5 μg/mL ascorbic acid 2-phosphate (Sigma), and 10 mM/L β-glycerolphosphate (Sigma) [34]. To observe calcium deposition, cultures were stained with Alizarin Red S (Nuclear). To induce adipogenic differentiation, MSCs were cultured with 10–8 M dexamethasone (Sigma), 2.5 μg/mL insulin, and 50 μg/mL indomethacin (Sigma) [35]. Adipocytes were easily discerned from undifferentiated cells by phase-contrast microscopy. To further confirm their identity, cells were fixed with 4% paraformaldehyde with PBS and stained with Oil Red (Sigma) on day 21 of adipogenic differentiation. To induce chondrogenic differentiation, MSCs were cultured in DMEM supplemented with 10 ng/mL TGF-β1 (Sigma), 50 nM ascorbic acid 2-phosphate (Sigma), and 6.25 mg/mL insulin for 3 weeks. To confirm differentiation, cells were fixed with 4% paraformaldehyde in PBS for 1 h at room temperature and stained with Alcian Blue pH 2.5 for detecting chondroblast secreted cell matrix (with chondrogenic differentiation) (data not shown).

Flow cytometry was performed in a FACSCalibur system (Becton Dickinson), using commercially available antibodies and following standard procedures. Cells were plated in 96-well plates and then centrifuged for 4 min at 300×*g*, 4 °C. Then, 10 μL of anti-CD16/32 antibody were added to wells (Fc receptor blocker), remaining for 15 min at 4 °C. At the end of incubation, the cells were washed with PBS (100 μL/well), the plate was centrifuged at 300×*g* for 4 min at 4 °C, and the supernatant was discarded. Subsequently, the cells were incubated with 10 μL of solution containing anti-CD11b/FITC, anti-Sca-1/APC, anti-CD44/Pecy5, anti-CD45/PE, anti-CD49/PE, and anti-CD34/EFluor660 antibodies for 30 min at 4 °C under light protection. Then, the cells were washed with 100 μL of PBS and again centrifuged at 300×*g* for 4 min at 4 °C. After discarding the supernatant, the cells were resuspended in 200 μL of 1% PFA and transferred to FACS tubes containing 100 μL of PBS. Data were analyzed using FlowJo software, version 8.7. The respective antibody isotypes of the abovementioned antibodies or samples of unlabeled cells were used as controls. An undifferentiated population of MSCs was used in all experiments.

### Peritoneal washing

The C57BL/6 mice were anesthetized with sevoflurane (Sevorane®, Abbott) and euthanized by terminal anesthesia with CO_2_. Washing of the peritoneal cavity was performed through a small incision in the cavity where 5 mL of RPMI-1640 (Sigma) was injected at 4 °C with the aid of a 24G needle and 5-mL syringe. After homogenizing the liquid inside the animal’s peritoneal cavity, the injected RPMI-1640 (Sigma) was collected with the aid of a syringe and needle into a 15-mL tube and placed on ice (4 °C), to prevent cell adhesion to the tube. Shortly afterwards, the recovered volume was centrifuged for 5 min at 1500 rpm, 4 °C. The pellet was resuspended in 5 mL of RPMI-1640 (Sigma) without SFB, and the total cell count was obtained in a Neubauer chamber. 5 × 10^5^ macrophages were plated in each well of the 24-well plate and, after 1 h (necessary for the cells to adhere to the plate surface), the RPMI without FBS was removed, the cells were washed 3 times with PBS at 37 °C, and 500 μL of RPMI-1640 (Sigma) with 10% FBS (Invitrogen) was added to each well. After 24 h, wells were washed with PBS at 37 °C to purify the culture, removing the B1 lymphocytes and leaving only the macrophages.

### In vitro macrophage infection

The plated macrophages were infected with 2.5 × 10^6^ *Leishmania amazonensis* per well (5:1 ratio) for 4 h. After this period, the macrophages were washed with PBS at 37 °C to remove any parasites that were not phagocytosed. Then, 500 μL of RPMI-1640 (Sigma) 10% fetal bovine serum (Cultilab) was added to the wells that did not receive co-culture, and 400 μL of RPMI-1640 (Sigma) 10% fetal bovine serum (Cultilab) to those that would receive co-culture later.

### Co-culture of macrophages with MSCs

After infection with *Leishmania amazonensis*, 100 μL of RPMI-1640 (Sigma) 10% fetal bovine serum (Cultilab) containing 5 × 10^4^ MSCs were added and left for 48 h. The plate was washed 3 times with PBS at 37 °C, and the slides were stained with a Rapid Panoptic kit (LaborClin).

### Macrophage and amastigote count

Panoptic-stained macrophages and MSCs were counted in groups of 50 cells per slide, under light microscopy (Olympus, CX31), at × 100 magnification. Then, the percentage of infected cells, the mean of intracellular amastigotes per macrophage, and the total amount of amastigotes present in the slides were calculated. Representative images were obtained using a bright-field microscope (Olympus BX51) coupled to a digital camera (Olympus DP72) at × 1000 magnification with the aid of the Cell F 3.1 software program (Olympus).

### Wound healing assay

To perform the wound healing assay, 3 × 10^4^ 3 T3 fibroblast cells were plated per well in a 96-well plate (TPP) and cultured with DMEM + 10% FBS at 37 °C and 5% CO_2_ until the monolayer of cells reach 90% of confluence. The culture medium was then changed to DMEM + 5% FBS to minimize cell proliferation, but keep it sufficient to prevent apoptosis and/or cell detachment.

After 24 h, the wells were scratched, washed 3 times with PBS, and the culture mediums added. To evaluate the groups, each well received 50% RPMI + 50% conditioned medium from the infected macrophage cultures. The plates were placed in an Incucyte® ZOOM live-cell analysis system (Essen BioScience) and photographed every 20 min for 96 h. Cell migration was analyzed in IncuCyte® ZOOM 2015A software (Essen BioScience).

### In vivo infection

After being received from the UFF vivarium, the animals were infected by subcutaneous injection of 20 μL PBS containing 2 × 10^6^ promastigotes of *Leishmania amazonensis* into the right hind footpad.

### Mesenchymal stromal cell treatment

The transplanted cell dosage was based on pilot studies (data not shown). For this purpose, different doses of AD-MSCs and BM-MSCs have been tested and the lowest effective dosage was chosen to avoid possible side effects associated with higher doses (e.g., embolism). The mice received two doses of 1 × 10^5^ AD-MSCs or BM-MSCs to treat the infection, the first 15 days after infection and the second 21 days post infection. For instillation, the cells were detached from the culture bottles with 0.25% trypsin and resuspended in culture medium in 15-mL conical polystyrene tubes. The tubes were centrifuged at 1200 RPM for 5 min at room temperature, and the cell pellet was resuspended in 1 mL of saline (NaCl 0.9%). The quantification and determination of cell viability was performed by removing a 10-μL aliquot of cells, subsequently diluted in 10 μL of Trypan Blue (0.02%) (Sigma). From this dilution, 10 μL of cell suspension was removed and placed in a Neubauer chamber (Hausser Scientific) for cell counting. The number of viable cells was counted in the four quadrants and the appropriate dilution was obtained for the preparation of injections of 50 μL of saline with 10^5^ cells in suspension.

### Administration of AD-MSCs or BM-MSCs

AD-MSCs or BM-MSCs were injected into the jugular vein, at a dose of 1 × 10^5^. Animals were anesthetized with sevoflurane (Sevorane®, Abbott), and the left jugular vein was dissected. A 0–5-mL syringe and an insulin needle (0.3 × 13 mm) were used to inject the cells in the cephalocaudal direction, and the vein was clamped for a few seconds to avoid any loss of blood or injected cells.

### Administration of meglumine antimoniate

Treatment was begun on the 18th day after infection. On alternate days, the animals received 2 mg of meglumine antimoniate (100 mg/kg) in 100 μL of PBS, at a concentration of 20 mg/mL. The drug was injected intraperitoneally for 36 days.

### Clinical profile (lesion progression and parasite load)

Lesion growth was monitored weekly with precision calipers (Mitutoyo). The measurement of the uninfected footpad was then deducted. On the predefined day after infection, mice were euthanized and the infected footpad of each animal was removed, placed in a 70% alcohol solution for 1 min, and individually weighed. Additionally, the draining popliteal lymph node was removed and placed in a vial with 1 mL of M199 medium. Footpad homogenates were obtained by manual maceration of the lesions with addition of 1 mL M199 medium and centrifuged for sedimentation of the heavier particles. The number of parasites in the footpads was determined by the limiting dilution assay (LDA). The macerates were diluted into 96-well culture plates (Jet Biofil, China) and incubated at 26 °C for 15 days. Promastigote cultures were observed with an optical microscope (Olympus, Japan), and the last well containing promastigotes was observed and noted.

### Detection of markers by flow cytometry

The cellularity of the popliteal lymph node macerate was quantified by counting in a Neubauer chamber (1 × 10^6^ cells per well in 96-well plates) to characterize T CD4^+^ cells and T_regs_ (CD4^+^, CD25^+^, FoxP3^+^). The plates were centrifuged at 300×*g* for 4 min; 100 μL of FACS buffer (PBS in 10% FBS) was added to the pellet and the cells were centrifuged again.

For characterization of T_regs_, non-specific-labeling blockade with anti-FCR (1:100) incubation was done for 5 min, followed by surface labeling with anti-CD3/Pacific Blue (1:200), anti-CD4/Pecy7 (1:200), anti-CD8/FITC (1:200), and anti-CD25/APC (1:200) antibodies for an additional 30 min. The volume was made up to 200 μL in FACS buffer; the cells were centrifuged, followed by fixation in kit fixation buffer (eBioscience) for 1 h. Then, the cells were washed and 100 μL of permeabilization buffer (eBioscience) was added to the pellet and incubated for 1 h, followed by centrifugation. The cells were then incubated with anti-FCR for 20 min and intracellular labeling was performed with anti-FoxP3/AlexaFluor 488 (1:50) for 30 min. The cells were washed in permeabilization buffer and resuspended in FACS buffer for flow cytometry analysis in a FACSCalibur system. The results were analyzed in FlowJo 8.7 software.

### Detection of cytokines by enzyme-linked immunosorbent assay (ELISA)

Concentrations of the cytokines present in cell culture supernatants and the footpad homogenate supernatants were determined by the ELISA using commercial kits (BD OptEIA) according to the datasheet instructions. Recombinant murine TNF-α, VEGF, IL-10, IFN-γ, and IL-4 were used to generate the standard curves. The minimum detection limits for these tests are 31.3 pg/mL for VEGF and IL-10 and 15.72 pg/mL for TNF-α, IFN-γ, and IL-4.

### Statistical analysis

No formal sample size calculation was performed to determine the numbers of animals per group; the sample size was based on the experience of our laboratory in previous studies using this model of cutaneous leishmaniasis. All results were analyzed in GraphPad Prism v6.0 (GraphPad Software). The statistical significance of the differences between groups was determined by two-way ANOVA (for lesion progression and wound healing) followed by Dunn’s test or one-way ANOVA (for all other comparisons) followed by Tukey’s test. The values are expressed as mean ± standard deviation.

## Results

### Co-culture of *Leishmania amazonensis*-infected macrophages with BM-MSCs, but not with AD-MSCs, resulted in lower nitric oxide production and higher parasite load

**Fig. 1:**
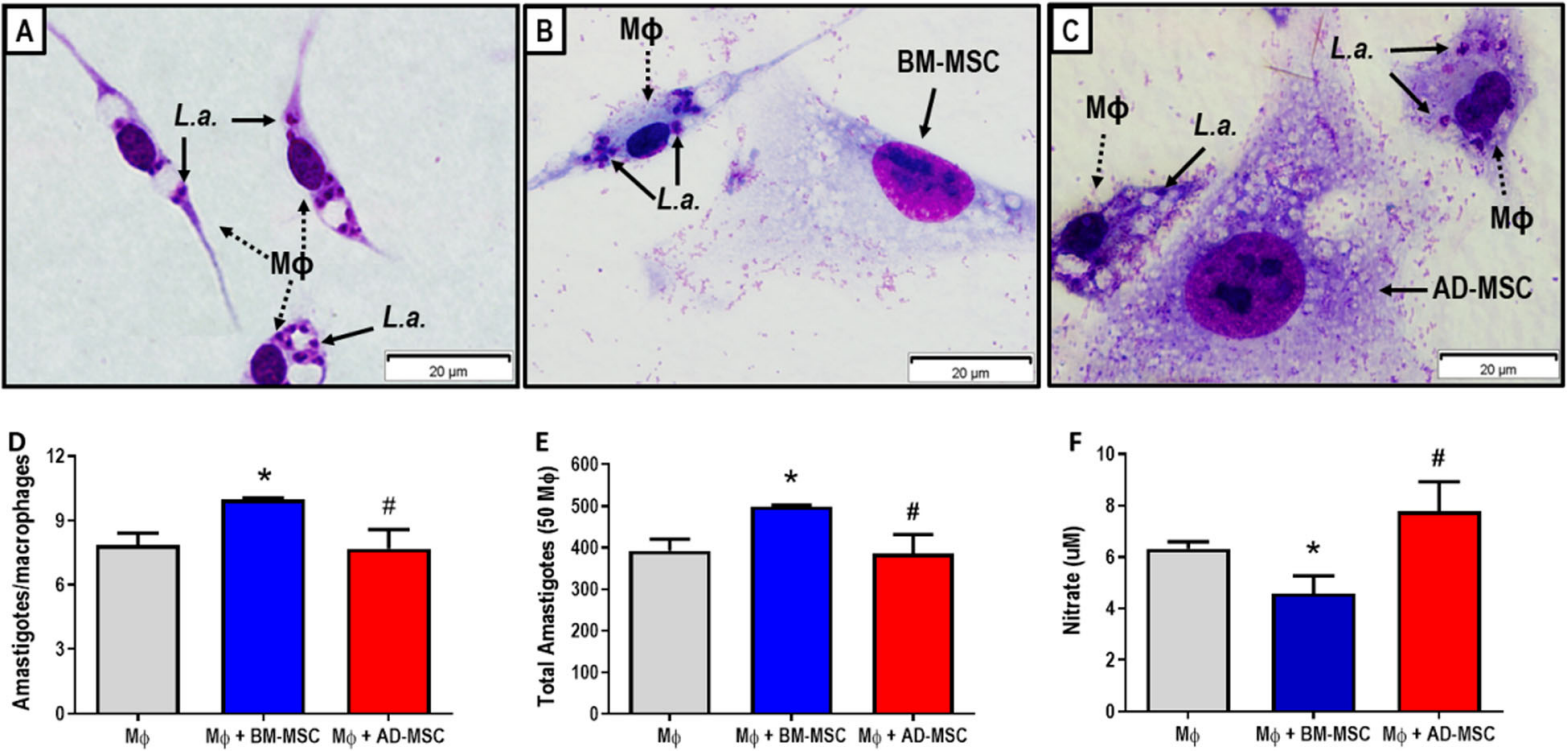
In vitro infection of C57BL/6-derived peritoneal macrophages in co-culture with bone marrow (BM)-derived mesenchymal stromal cells (MSCs) or adipose tissue (AD)-derived MSCs. Representative images of peritoneal macrophages after infection with *Leishmania amazonensis* and macrophages and amastigote counts under the microscope. **a** Culture of infected macrophages. **b** Co-culture of infected macrophages with BM-MSCs. **c** Co-culture of infected macrophages with AD-MSCs. **d** Total amastigotes per macrophage. **e** Total amastigotes per well. **f** NO measurement. Rapid panoptic stain. Scale 20 μm. (*N* = 2) Representative of two independent experiments. Values show the mean ± standard deviation. *Significantly different from infected Mf without co-culture (control) (*P* < 0.05). ^#^Significantly different from infected Mf treated with BM-MSC (*P* < 0.05)

To assess whether MSCs could alter the leishmanicidal capacity of C57BL/6 macrophages, we infected macrophages with *Leishmania amazonensis* and co-cultured them with BM-MSCs and AD-MSCs for 48 h. The cells were then stained (Fig. 1a–c) and the parasite load quantified. The presence of BM-MSCs increased the parasite load (total number of amastigotes) in C57BL/6 macrophages. However, the presence of AD-MSCs in the culture did not alter the parasite load per macrophage (Fig. 1d). The total number of amastigotes per well behaved similarly (Fig. 1e).

There was also a reduction in the nitric oxide concentration in infected macrophages co-cultured with BM-MSCs compared to macrophages not co-cultured with MSCs co-culture and those co-cultured with AD-MSCs (Fig. 1f). These findings suggest that BM-MSCs induce macrophage susceptibility to *Leishmania* infection, which is likely associated with lower NO production, while the AD-MSCs do not affect the susceptibility of the macrophages.

### Treatment with AD-MSCs promoted partial protection against lesion progression after *Leishmania amazonensis* inoculation in vivo

**Fig. 2:**
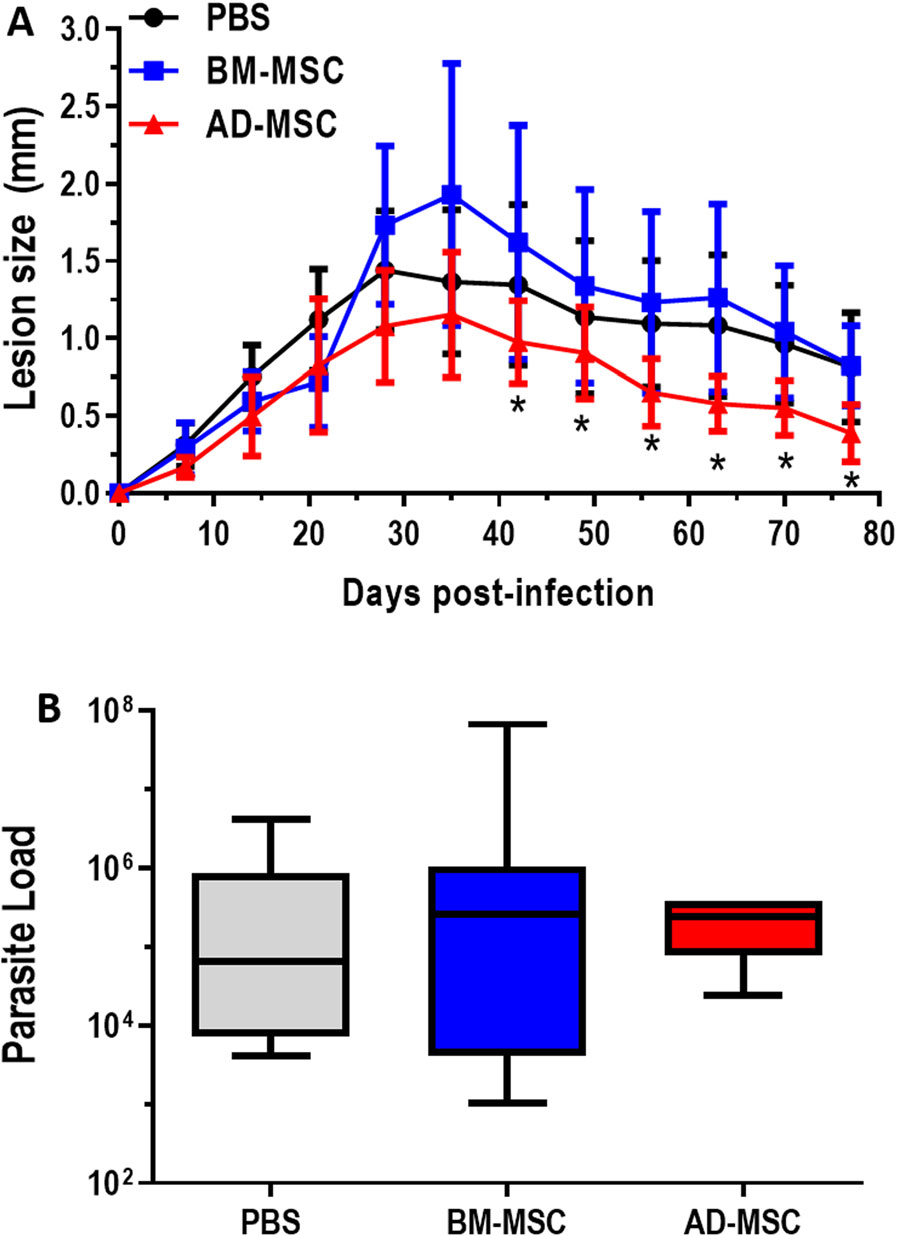
Effect of BM-MSC and AD-MSC treatment on lesion progression in infected C57BL/6 mice. C57BL/6 mice were inoculated in the footpad with *Leishmania amazonensis*; treated either with PBS, BM-MSC, or AD-MSC; and had lesion development measured with calipers on a weekly basis. After 77 days, the mice were euthanized, and the parasite load of the paw was determined by limiting dilution. **a** Analysis of lesion progression. **b** Analysis of parasite load in the paw. Representative of four independent experiments. *N* = 8. Values show the mean ± standard deviation. *Significantly different from PBS (control group) (*P* < 0.05)

To evaluate whether treatment with BM-MSCs or AD-MSCs would be effective in an in vivo infection, C57BL/6 mice were infected with *Leishmania amazonensis* and treated with two doses of BM-MSCs or AD-MSCs intravenously, as described in the “Methods” section. AD-MSCs, but not BM-MSCs, were associated with less lesion progression compared to untreated control (Fig. 2a). However, treatment with MSCs (regardless of source) was not associated with any significant differences in parasite load 77 days after infection, when compared to control (Fig. 2b). These findings indicate that therapy with BM-MSCs is not beneficial in this model; however, AD-MSCs are able to partially control lesion progression.

### Supernatant of infected macrophages cultured with AD-MSC-conditioned medium induces faster healing

**Fig. 3:**
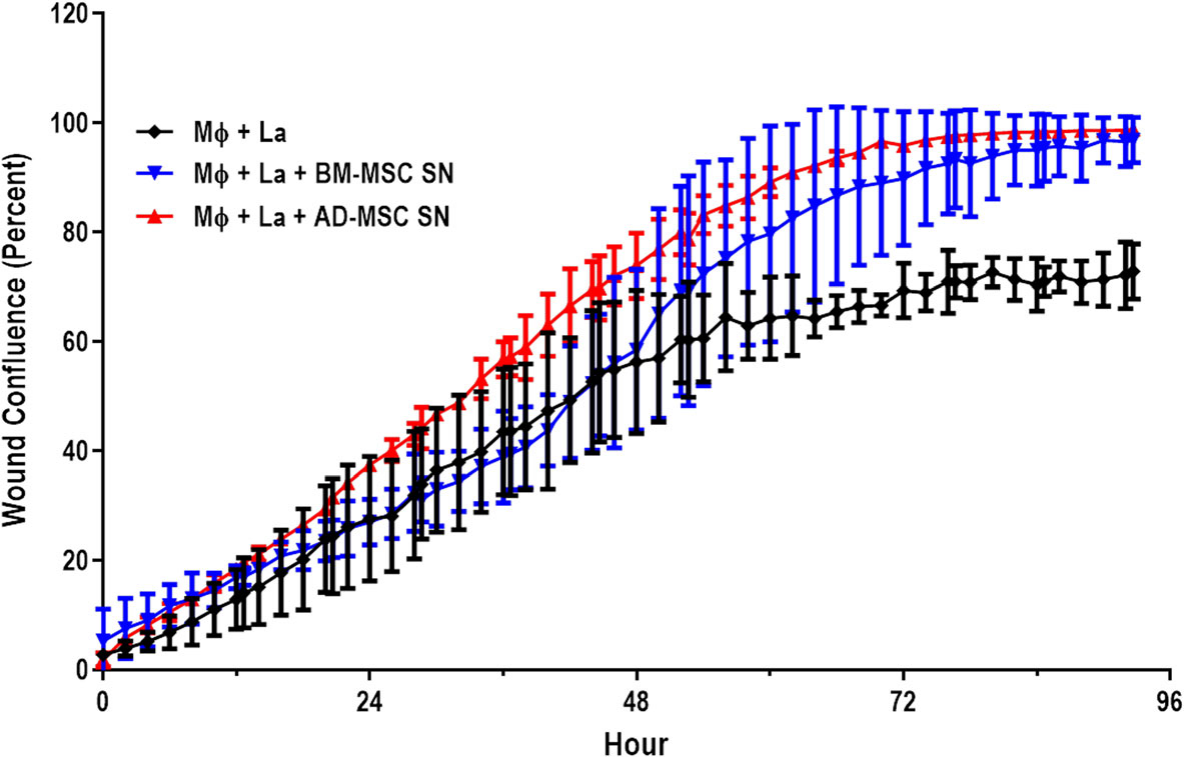
Wound healing scratch assay. 3T3 cells were cultured until reaching 80% confluence. The cell monolayers were scratched, and the medium of the culture was changed as follows: RPMI (negative control—Supp, Fig. 1); DMEM + FBS (positive control—Supp, Fig. 1); RPMI + supernatant of infected macrophages (Mɸ + La); RPMI + supernatant of infected macrophages cultured with conditioned medium of MSCs (Mɸ + La + AD-MSC SD and Mɸ + La + BM-MSC SD). The scratch on the cell monolayer was photographed every 20 min for 96 h and analyzed by IncuCyte ZOOM 2015A. Values show the mean ± standard deviation

To understand the partial protection induced by treatment with AD-MSCs in vivo, a scratch wound healing assay was performed. 3T3 cells (fibroblast cell line) were cultured until 80% confluence, and the cell monolayer was scratched. The culture medium was switched out for either RPMI + supernatant of infected macrophages (Mɸ + La) or RPMI + supernatant of infected macrophages cultured with conditioned medium of MSCs (Mɸ + La + AD-MSC SD and Mɸ + La + BM-MSC SD). The wells where photographed every 20 min for 96 h. We observed that cells treated with RPMI + supernatant of macrophages cultured with AD-MSC conditioned medium exhibited faster healing than wells treated with RPMI + supernatant of macrophages cultured with BM-MSC-conditioned medium (Fig. 3). We used a negative control and a positive control in the experiment (Additional file 1: FigureS1). This suggests that the molecules produced by AD-MSCs in contact with the infected macrophages induce faster healing.

### Co-culture of infected macrophages with AD-MSCs increased the production of TNF-α and IL-10

**Fig. 4:**
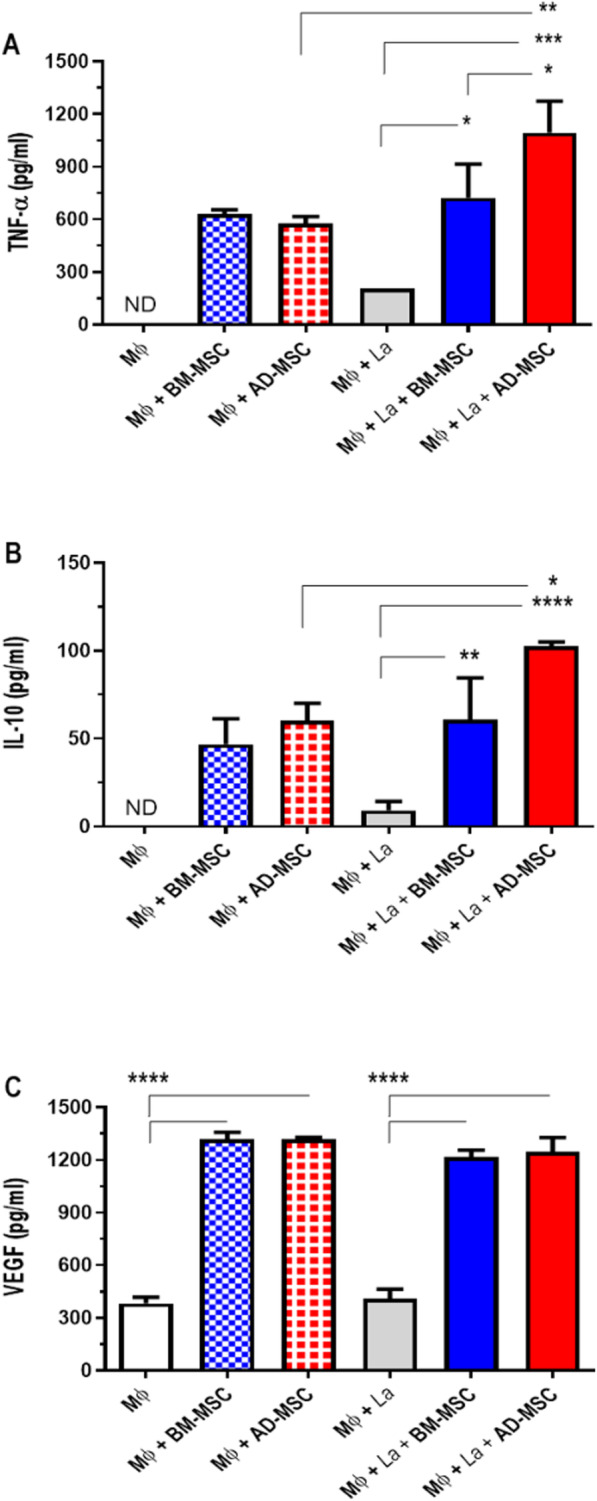
Analysis of cytokine concentrations in cell culture supernatants. Concentrations of the cytokines TNF-α (**a**), IL-10 (**b**), and VEGF (**c**) present in supernatants of cell cultures were determined by enzyme-linked immunosorbent assay (ELISA) using commercial BD kits, according to the manufacturer’s instructions. Groups: Macrophages (Mɸ); macrophages co-cultured with BM-MSCs (Mɸ + BM-MSC); macrophages co-cultured with AD-MSCs (Mɸ + AD-MSC); macrophages infected with *Leishmania amazonensis* (Mɸ + La); macrophages infected with *Leishmania amazonensis* co-cultured with BM-MSCs (Mɸ + La + BM-MSC); macrophages infected with *Leishmania amazonensis* co-cultured with AD-MSCs (Mɸ + La + AD-MSC). Representative of two independent experiments. *N* = 3. Values show the mean ± standard deviation. *P < 0.05, **P < 0.01, ***P < 0.005, and ****P < 0.001 indicate a significant difference between the groups. ND not detected

Next, the co-culture supernatants were evaluated by ELISA to determine the effects of MSCs on macrophages. Co-culture of BM-MSCs and AD-MSCs with infected macrophages increased the production of TNF-α (Fig. 4a), IL-10 (Fig. 4b), and VEGF (Fig. 4c) in comparison to non-infected macrophages without MSC co-culture. However, co-culture of infected macrophages with AD-MSCs was associated with an increase in the production of TNF-α and IL-10 (Fig. 4a, b) in relation to AD-MSCs with non-infected macrophages and BM-MSC co-culture with both non-infected and infected macrophages. The infection did not modulate VEGF production. These results suggest that co-culture of macrophages with AD-MSCs makes the macrophages more responsive to infection, which could enhance the immune response and thus correlate with lesion control.

### Combination therapy with meglumine antimoniate and AD-MSCs conferred superior protection against *Leishmania amazonensis* infection in vivo

**Fig. 5:**
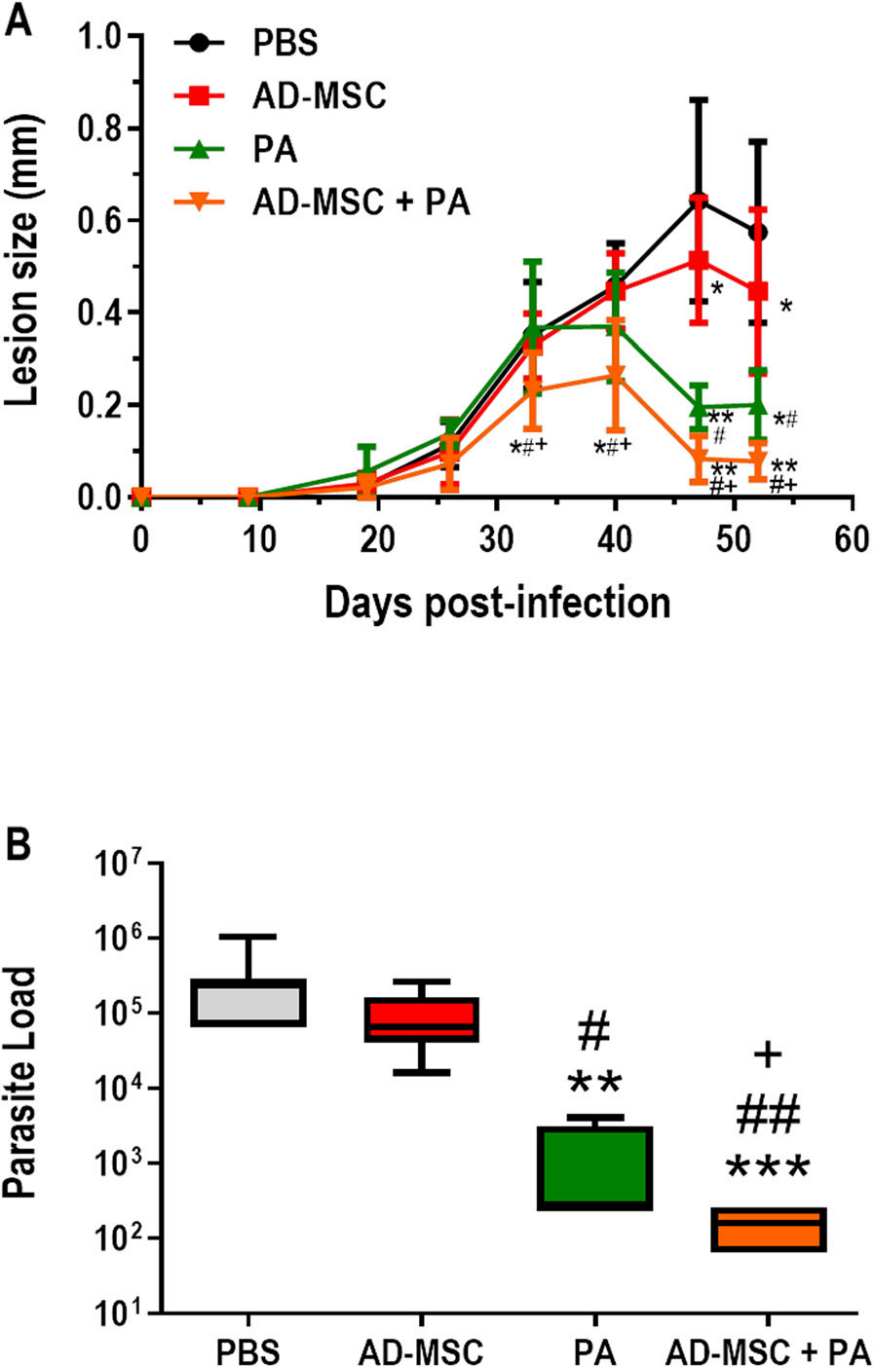
Effect of combined therapy with AD-MSCs and a pentavalent antimonial on clinical profile. C57BL/6 mice were inoculated in the footpad with *Leishmania amazonensis*. On days 15 and 21 post-infection, the AD-MSC and AD-MSC + PA groups received AD-MSC and the control group was injected with PBS. On day 18, the PA and AD-MSC + PA groups started treatment with meglumine antimoniate, administered on alternate days until day 50 post-infection. After 52 days, the mice were euthanized and the parasite load was determined by limiting dilution. **a** Analysis of lesion progression. **b** Analysis of parasite load in the footpad. **c** Analysis of parasite lode in the popliteal lymph node. *N* = 9 in the PBS group and *N* = 6 in the other groups. Values show the mean ± standard deviation. *P < 0.05, **P < 0.01, and ***P < 0.005 indicate a significant difference between the groups in relation to the control (PBS); ^#^*P* < 0.05 and ^##^*P* < 0.01 indicate a significant difference between the groups in relation to the AD-MSC; ^+^*P* < 0.05 indicates a significant difference in the AD-MSC group in relation to the PA group

As AD-MSCs conferred partial protection against injury and lesion progression, but did not affect parasite load in vivo, we combined cell therapy with meglumine antimoniate (pentavalent antimonial, PA) treatment to assess whether combination therapy would be more effective. *Leishmania amazonensis*-infected C57BL/6 mice were separated into 4 groups (PBS; AD-MSC; PA; AD-MSC + PA). On days 15 and 21 post-infection, the mice received doses of AD-MSCs. On the 18th day after infection, the treatment with pentavalent antimonial was initiated and the mice continued to receive the drug on alternate days. Treatment with AD-MSC reduced lesion size in comparison with PBS, but no significant difference was observed in parasite load. Mice treated with PA reduced lesion size and parasite load, as expected, in comparison to PBS. However, mice treated with combined therapy (PA + AD-MSC) presented a further reduction of both lesion size (Fig. 5a) and parasite load (Fig. 5b) compared to all other groups. These results indicate that the combination of AD-MSCs and PA controls lesion development and parasite load better than AD-MSCs or PA alone.

### Mice treated with AD-MSCs and meglumine antimoniate exhibited a reduced inflammatory response

**Fig. 6:**
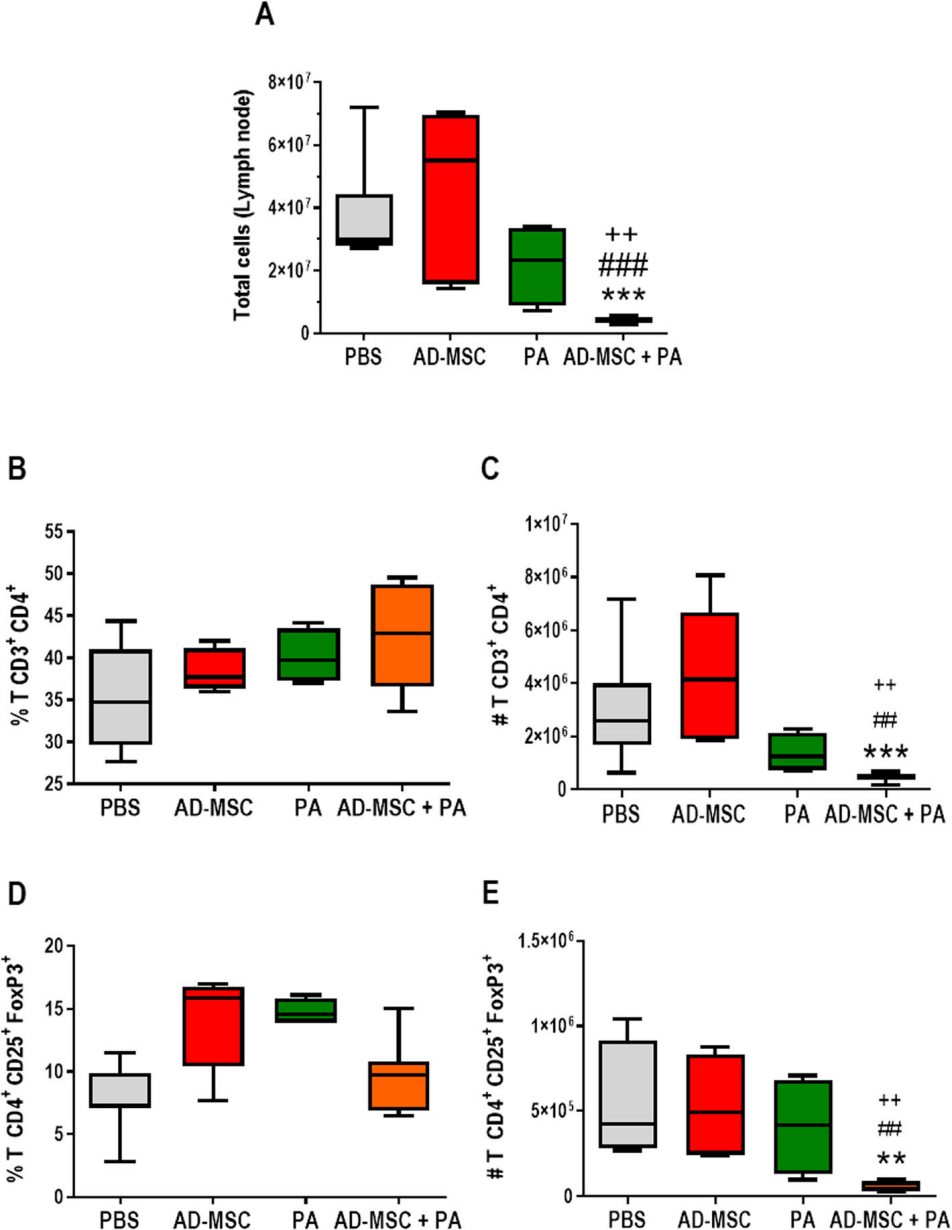
Lymph node cell count and detection of T CD4^+^ and T_reg_ lymphocytes by flow cytometry (FACS). Cells were collected from the draining popliteal lymph node macerate, counted under a microscope (× 4 magnification), and analyzed by flow cytometry (FACS CANTO BD) for T CD4^+^ and T_reg_ (CD4^+^ CD25^+^ FoxP3^+^) lymphocyte expression after 52 days of infection. Results shown as percentage and total population of cells positive for these markers in CD3^+^ marker-positive lymphocytes. **a** Total number of lymph node cells. **b** Percentage of CD4^+^ T lymphocytes. **c** Total CD4^+^ T lymphocyte population. **d** Percentage of T_reg_ lymphocytes. **e** T_reg_ lymphocyte population. Values show the mean ± standard deviation for each group. *P < 0.05 **, *P* < 0.01, and ***P < 0.005 indicate a significant difference between the groups in relation to the control (PBS); ^#^*P* < 0.05, ^##^*P* < 0.01, and ^###^*P* < 0.005 indicate a significant difference between the groups in relation to the AD-MSC; ^+^*P* < 0.05 indicates a significant difference in the AD-MSC group in relation to the PA group

**Fig. 7:**
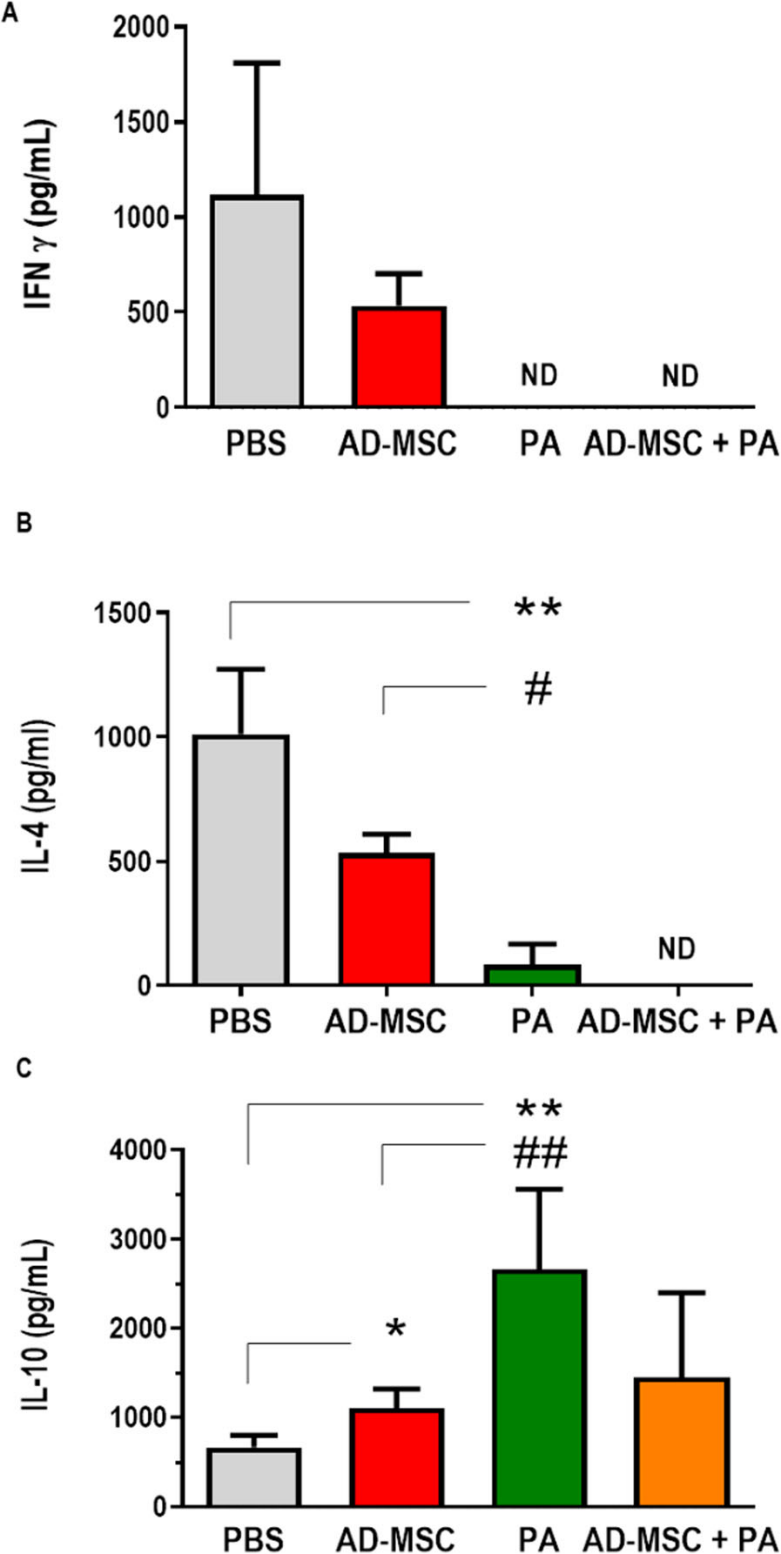
Analysis of cytokine concentrations at the site of infection. Concentrations of the cytokines IFN-γ (**a**), IL-4 (**b**), and IL-10 (**c**) present in supernatants of paw homogenates were determined by enzyme-linked immunosorbent assay (ELISA) using commercial BD kits, according to manufacturer instructions.*P < 0.05 and **P < 0.01 indicate a significant difference in relation to the control (PBS); ^#^*P* < 0.05 and ^##^*P* < 0.01 indicate a significant difference between groups in relation to the AD-MSC group. ND not detected

Based on the capacity of MSC therapy to modulate the T cell response and population, we investigated the population of lymphocytes present in the draining lymph node. Lymph node cellularity was reduced in the group receiving combination treatment in comparison to the other groups (Fig. 6a). Although there were no significant differences in the percentages of CD4^+^ T cells (Fig. 6b) and T_regs_ (CD4^+^ CD25^+^ FoxP3^+^) (Fig. 6d) between groups, the reduced total cell number meant significantly lower numbers of CD4^+^ T cells and T_regs_ (Fig. 6c, e). This result suggests a decreased inflammatory response in the animals receiving combination therapy. We also evaluated the cytokine profile in the lesion. Neither IFN-γ (Fig. 7a) nor IL-4 (Fig. 7b) were detected in the PA and AD-MSC + PA groups, indicating resolution of infection. IL-10 was significantly lower in the PBS control group compared to the treated groups (Fig. 7c). These results indicate a faster healing process in the infection in all treated groups, but particularly in the group that received combination therapy.

## Discussion

The discovery of treatments that can combat both the parasite and the immunopathology caused by leishmaniasis would greatly improve patient quality of life. Several studies have demonstrated the immunomodulatory role of MSCs in the control of inflammation. Thus, we hypothesized that MSC therapy could aid in the resolution of leishmaniasis through immunomodulation of the infection site and restoration of tissue homeostasis. MSCs have an immunomodulatory effect when used in the pro-inflammatory phase of sepsis, increasing the phagocytic capacity of immune cells [8] and causing macrophages of septic mice to produce more IL-10, shifting to a more anti-inflammatory phenotype, as well as improving organ function, which promotes a higher rate of survival [22]. In parasitic diseases, MSCs have been shown to decrease tissue inflammation in infections with *Schistosoma japonicum* [36] and *Trypanosoma cruzi* [14, 15], as well as decreasing parasitemia [16]. Furthermore, they have been shown to polarize macrophages towards a regulatory phenotype, resulting in increased levels of IL-10 and IL-12 [37].

*Leishmania amazonensis* causes much less activation and production of cytokines by immune cells than do other species of *Leishmania* [18, 19]. C57BL/6 mice infected with *Leishmania amazonensis* display a progressive phase of infection followed by partial resolution and chronic infection [38]. This closely resembles the profile observed in cases of human cutaneous leishmaniasis, which is typically a self-limiting disease [39, 40]. Parasites can still be found in healed lesions [40].

Although phenotypically similar, MSCs obtained from different tissues have different effects when used therapeutically, as demonstrated in studies of emphysema [30], sepsis [41], asthma [42], and bone regeneration [43]. Therefore, we investigated the effects of both bone marrow- and adipose tissue-derived MSCs. To understand the potential influence of MSCs on macrophages infected with *Leishmania amazonensis*, different co-cultures were performed. Co-culture with AD-MSCs had no effect on in vitro infection (Fig. 1d, e). This has also been observed for *Leishmania major* infection, where C57BL/6 peritoneal macrophages exposed to AD-MSCs through transwell plates and then infected did not present any difference in the number of amastigotes compared to a control group without AD-MSC exposure [44]. In this study, however, infected macrophages co-cultured with BM-MSCs displayed greater infection (Fig. 1d, e). This is in keeping with previous observations of our group in macrophages from BALB/c mice [27]. This result could be explained by the reduction in NO induced by this co-culture (Fig. 1f), as NO is directly involved in the killing of *Leishmania* parasites and therefore is a key molecule in the control of the disease. Less NO production will thus decrease the leishmanicidal action of macrophages [45, 46]. Another factor that can be related to the differences observed in parasite load is the production of cytokines. Co-culture with AD-MSCs increased production of TNF-α and IL-10 as compared to co-culture with BM-MSCs (Fig. 4a and b). Thus, the two types of stromal cells had different effects on *Leishmania amazonensis*-infected macrophages, corroborating previously published work that has already shown that MSCs derived from different tissues produce different factors and effects [30, 42, 43, 47].

The success of wound healing has been associated with the resolution of inflammation. Chronic inflammation can lead to poor healing outcomes [48]. Therefore, the ability of MSCs to modulate the inflammatory response in wounds supports their favorable effect on the healing response. MSCs also enhance wound healing through paracrine effects, increasing the migration and proliferation of keratinocytes and fibroblasts [49, 50] and accelerating wound closure. In our study, AD-MSCs induced faster wound healing than BM-MSCs (Fig. 3). Our data are in accordance with Liu et al. [51] and Pelizzo et al. [52], who observed beneficial effects of AD-MSCs on wound closure, as a result of better re-epithelialization and thickening of granulation tissue, when compared to BM-MSCs.

Adipose-derived stromal vascular fraction cells (AD-SVFs) are a cellular extract, rich in AD-MSCs, which can be easily obtained from adult (especially human) fat tissues [53]. AD-MSCs and AD-SFVs have been used successfully in several clinical applications, such as breast reconstruction [54], cartilage repair [55], androgenetic alopecia [56], atopic dermatitis [57], cutaneous wound healing [58], skin scars [59, 60], and modulation of cancer growth by repairing wounded tissue [61].

The effects of MSCs may differ depending on the route of administration. In previous work by our group, BALB/c mice with leishmaniasis were treated with intravenous and intralesional administration of BM-MSCs. Only the intravenous route resulted in beneficial effects on the immune response of infected animals, with a decrease in IFN-γ-producing T CD8^+^ cells and an increase in IL-10-producing T CD4^+^ and T CD8^+^ populations, in addition to an increase in the population of T_reg_ cells [27]. This T_reg_ cell population has been shown to be essential for the control of *Leishmania amazonensis* infection in B6 mice [26]. Although our previous study with BALB/c mice yielded negative results for BM-MSC treatment, we assessed both BM-MSCs and AD-MSCs in the C57BL/6 model. However, while AD-MSC treatment was able to decrease lesion progression, conferring partial protection, treatment with BM-MSCs was again ineffective in this murine cutaneous leishmaniasis model (Fig. 2a). However, neither treatment managed to reduce parasite load in vivo (Fig. 2b). Thus, AD-MSCs appear to be more promising than BM-MSCs as a treatment for *Leishmania amazonensis* infection. AD-MSCs have also been demonstrated to have a beneficial effect in *Leishmania major* infection, inducing CD8^+^/CD4^+^ T lymphocytes [62].

Based on the positive effects observed for AD-MSCs, it was hypothesized that the combination of AD-MSCs with conventional treatment (meglumine antimoniate) would “fight the parasite on two fronts,” with PA killing the parasites and MSCs halting the inflammation caused by the infection. Indeed, the combination of the two treatments led to decreases both in lesion size (Fig. 5a) and in the number of parasites in the footpad (Fig. 5b). Although the group that received meglumine antimoniate alone did show reductions in lesion size and parasite load, mice that received combination therapy showed an even greater reduction in both parameters, which may be attributed to the beneficial effects of meglumine antimoniate in reducing the parasite load and AD-MSCs decreasing the inflammatory process and repairing the lesion through paracrine effects. The group treated with combination therapy also had a reduced number of total cells in the lymph node (Fig. 6a), which resulted in fewer CD4^+^ T cells (Fig. 6c), CD8^+^ T cells (Additional file 2: Figure S2), and T_regs_ (Fig. 6e).

The success of this therapy may be directly related to the reduced cellularity in the popliteal lymph node. RAG2^−/−^ mice infected with *Leishmania major* demonstrated susceptibility to infection due to the absence of CD4+ T lymphocytes [63, 64]. However, MHCII^−/−^, RAG2^−/−^, and SCID mice are resistant to *Leishmania amazonensis* infection, with no macroscopic lesions, minimal cellular infiltrate, and reduced tissue parasite load [17], which suggests that the CD4^+^ T lymphocytes in the C57BL/6 mice contribute to the development of the lesion in cutaneous leishmaniasis caused by *Leishmania amazonensis.*

MSCs have the ability to induce T_regs_ [23–25]. It is well established that these cells are essential for tissue homeostasis, lesion progression, and parasite load in the C57BL/6 model of *Leishmania amazonensis* infection [26]. MSCs could act as a potential niche for T_regs_, promoting their generation, recruitment, phenotype maintenance, and function [23, 65]. Unlike what was observed in the BALB/c mice treated with BM-MSCs [27], in the present investigation, we did not find an increase in T_regs_ in the AD-MSC treatment group. Furthermore, in the combined treatment group, which had less lesion progression and a lower parasitic load, the total number of T_regs_ was the lowest in relation to the other treatment groups (Fig. 6d). We believe that a lower parasite burden leads to less induction of T_regs_. Therefore, the mechanism underlying protection in the AD-MSC treatment group does not appear to be related to T_regs_.

Several studies have demonstrated that AD-MSC treatment can modulate the production of several cytokines. For example, AD-MSC treatment decreases IL-4 levels, ameliorating allergic airway inflammation in a mouse model of asthma [24] and increasing survival in mice with sepsis [41]. Mello [16] demonstrated that AD-MSC treatment in mice with *Trypanosoma cruzi* infection decreases IFN-γ levels in the heart, increasing protection against heart damage. The production of cytokines, such as IL-4, IFN-γ, and IL-10, plays a key role in the susceptibility or resistance to leishmaniasis. In *Leishmania major* infection, the model that is the major example of the Th1-/Th2-response dichotomy, IFN-γ, a Th1 cytokine, plays an essential role in the control of parasite growth [66, 67]. Mice deficient in IFN-γ fail to cure *Leishmania major* infection [68]. Pinheiro and Rossi-Bergmann [69] have shown their role of these cytokines in controlling disease progression in the late phase of *Leishmania amazonensis* infection. In addition, several studies have shown the importance of IFN-γ in stimulating cytotoxic mechanisms that promote parasite elimination [70, 71]. IL-4, however, is one of the major cytokines involved in induction of the Th2-type response, together with IL-13. IL-4 production is associated with susceptibility to *Leishmania major* infection [72, 73] and to *Leishmania amazonensis* infection [74]. Production of this cytokine inhibits expression of the β2 chain of the IL-12 receptor, leading to development of a Th2 response and, hence, susceptibility to infection [75]. In the present study, no production of IFN-γ (Fig. 7a) or IL-4 (Fig. 7b) was detected at the site of infection after treatment with either combination therapy or meglumine antimoniate alone. This suggests that the pro-inflammatory response had already resolved at the time of euthanasia in both groups, and is consistent with previous findings in Chagas disease [16] and sepsis [41]. However, in *Leishmania major* infection, an enhancement of the Th1 response was observed after treatment with AD-MSCs; the treated mice exhibited increases in IFN-γ and TNF-α production, which was unexpected [62].

IL-10 has several effects on cutaneous leishmaniasis, one of which is the capability to suppress NO production and leishmanicidal activity in macrophages, leading to the suppression of Th1 responses [76]. In mice infected with *Leishmania amazonensis*, IL-10 partially contributes to the generation of immunodeficient responses [77, 78], which is the main factor by which susceptibility is increased in mice co-infected with phlebotomine saliva [79]. However, the role of IL-10 in *Leishmania amazonensis* infection is still poorly understood. The data obtained by cytokine measurement in the present study suggests increased production of IL-10 in the groups which received combination therapy or the pentavalent antimonial alone (Fig. 7c). This elevated IL-10 production may be due to the resolution of inflammatory response at the site of infection; as both lesion progression and parasite load are already controlled by this point, its presence would be to decrease pro-inflammatory factors, such as IFN-γ.

Mesenchymal stromal cells derived from adipose tissue appear to be the more promising option for the treatment of *Leishmania amazonensis* infection in a murine model, as opposed to MSCs derived from the bone marrow. AD-MSCs conferred partial protection during lesion development, while BM-MSCs did not provide any benefits in terms of lesion progression or parasite load. Furthermore, in vitro, infected macrophages co-cultured with AD-MSCs did not have their infection worsened, unlike macrophages co-cultured with BM-MSCs. Our data for *Leishmania amazonensis* infection support the longstanding notion that, although there are no significant phenotypic differences among MSCs from different sources [80, 81], they do indeed elicit different host responses [30, 41, 42].

This study has some limitations that should be addressed. First, a specific model of leishmaniasis was used; thus, whether results would be similar in other models is unknown. Second, further in vivo studies combining MSC therapy with macrophages should be performed, even though some reports have demonstrated a good relationship between the in vitro immunomodulatory capacity of MSCs and their in vivo immunomodulatory capacity [82, 83].

In summary, the combination of cell therapy using AD-MSCs and chemotherapy with pentavalent antimonials seems to potentiate the healing process in an experimental model of cutaneous leishmaniasis. This combination was able to reduce lesion progression, parasite loads, cellularity in draining lymph nodes, and cytokine production. To our knowledge, this is the first time a combined regimen of cell therapy and conventional treatment has been investigated in a model of *Leishmania* infection. This novel line of therapy warrants further investigation.

## Conclusion

Taken together, our data suggest that mesenchymal stromal cells derived from adipose tissue are an alternative and feasible therapy for cutaneous leishmaniasis caused by *Leishmania amazonensis*, especially as an adjunct to conventional treatment with meglumine antimoniate. AD-MSCs conferred greater protection against injury by reducing parasite load, mitigating the inflammatory response, and accelerating the healing process. Its combination with a pentavalent antimonial constituted an effective dual therapy, treating both the parasitic infestation itself and the immunopathology caused by *Leishmania amazonensis*.

## Supplementary information

**Additional file 1 : Figure S1.** Wound healing scratch assay controls. 3 T3 cells were cultured until reaching 80% confluence. The cell monolayers were scratched, and the medium of the culture was changed as follows: RPMI (negative control); DMEM + FBS (positive control). Values show the mean ± standard deviation.

**Additional file 2 : Figure S2.** Detection of T CD8^+^ lymphocytes by flow cytometry (FACS). Cells were collected from the draining popliteal lymph node macerate, counted under a microscope (40× magnification), and analyzed by flow cytometry (FACS CANTO BD) for T CD8^+^ lymphocyte expression after 52 days of infection. Results shown as percentage and total population of cells positive for these markers in CD3^+^ marker-positive lymphocytes. (A) Percentage of CD8^+^ T lymphocytes; (B) Total CD8^+^ T lymphocyte population; Values show the mean ± standard deviation for each group. * *P* < 0.05, ** *P* < 0.01 indicate a significant difference between the groups in relation to the control (PBS); # P < 0.05, ## P < 0.01, ### *P* < 0.005 indicate a significant difference between the groups in relation to AD-MSC; + P < 0.05 indicates a significant difference in the AD-MSC group in relation to the PA group.

## Data Availability

The datasets during and/or analyzed during the current study are available from the corresponding author on reasonable request.

## Abbreviations

MSCs: Mesenchymal stromal sells
BM-MSCs: Bone marrow-derived mesenchymal stromal cells
AD-MSCs: Adipose tissue-derived mesenchymal stromal cells
NO: Nitric oxide
VEGF: Vascular endothelial growth factor
IL-10: Interleukin-10
TNF-α: Tumor necrosis factor-alpha
IL-4: Interleukin 4
IFN-γ: Interferon-gamma
ATP: Adenosine triphosphate
GTP: Guanosine triphosphate
Treg: Regulatory T lymphocyte
PBS: Phosphate buffer
DMEM: Dulbecco’s minimal essential medium
CO_2_: Carbon dioxide
CD: Cluster of differentiation
LDA: Limiting dilution assay
ELISA: Enzyme-linked immunosorbent assay
FACS: Fluorescence-activated cell sorting
T CD4^+^: CD4^+^ T lymphocyte
T CD8^+^: CD8^+^ T lymphocyte
Mɸ: Macrophages
PA: Pentavalent antimonial
IL-12: Interleukin-12
MHC: Major histocompatibility complex
IL-13: Interleukin-13
