# Supplementary material for: Combined therapy with adipose tissue-derived mesenchymal stromal cells and meglumine antimoniate controls lesion development and parasite load in murine cutaneous leishmaniasis caused by Leishmania amazonensis
Source: Stem Cell Res Ther. 2020 Aug 31;11:374. doi: 10.1186/s13287-020-01889-z (PMC7457509; doi:10.1186/s13287-020-01889-z)
Supplement: Supplementary file 1 — Additional file 1 : Figure S1. Wound healing scratch assay controls. 3 T3 cells were cultured until reaching 80% confluence. The cell monolayers were scratched, and the medium of the culture was changed as follows: RPMI (negative control); DMEM + FBS (positive control). Values show the mean ± standard deviation. [file 13287_2020_1889_MOESM1_ESM.pdf]

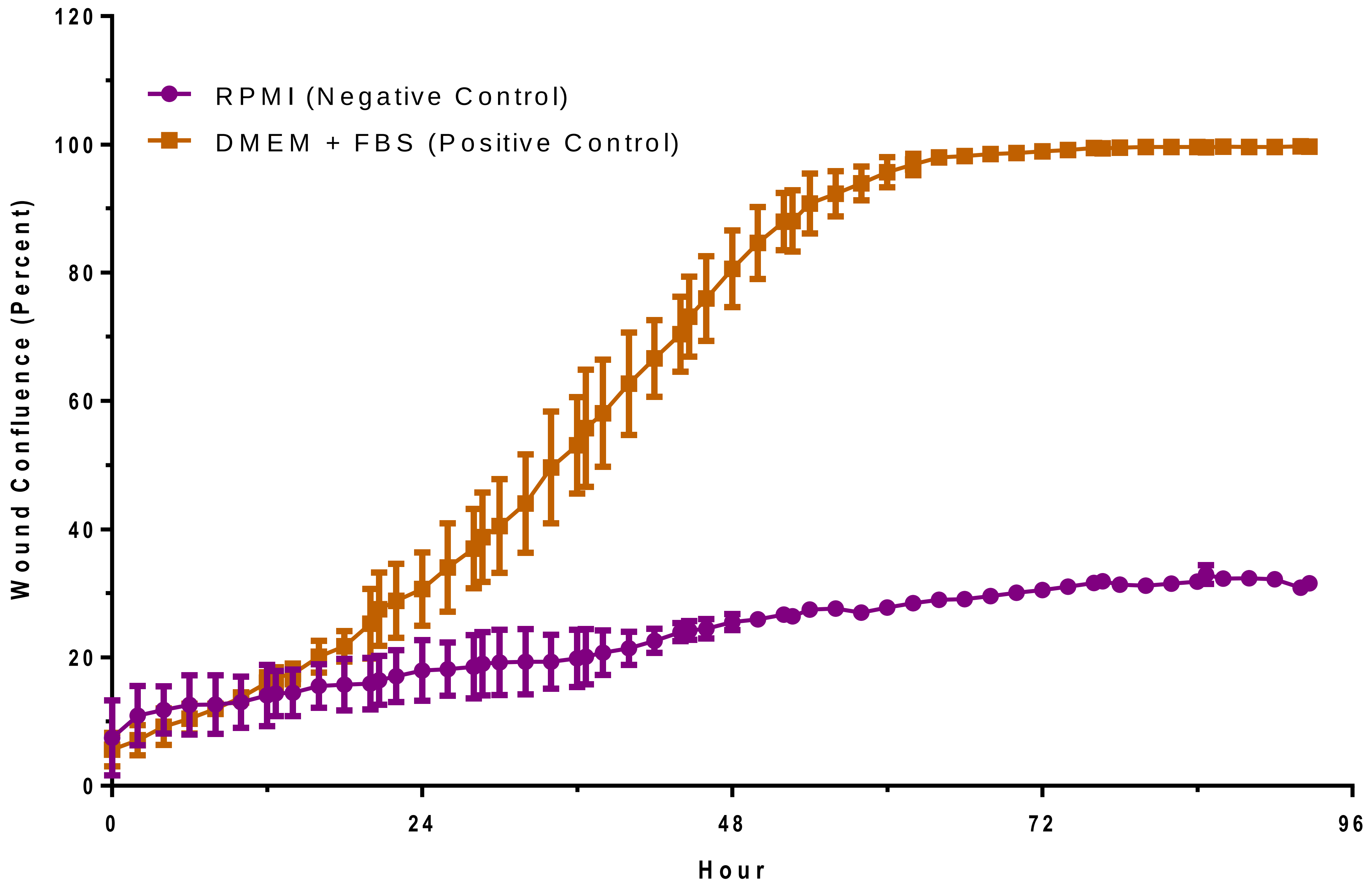

Additional File 1: Figure S1: Wound healing scratch assay controls. 3T3 cells were cultured until reaching 80% confluence. The cell monolayers were scratched, and the medium of the culture was changed as follows: RPMI (negative control); DMEM + FBS (positive control). Values show the mean ± standard deviation.
