# Supplementary material for: Combined therapy with adipose tissue-derived mesenchymal stromal cells and meglumine antimoniate controls lesion development and parasite load in murine cutaneous leishmaniasis caused by Leishmania amazonensis
Source: Stem Cell Res Ther. 2020 Aug 31;11:374. doi: 10.1186/s13287-020-01889-z (PMC7457509; doi:10.1186/s13287-020-01889-z)
Supplement: Supplementary file 2 — Additional file 2 : Figure S2. Detection of T CD8+ lymphocytes by flow cytometry (FACS). Cells were collected from the draining popliteal lymph node macerate, counted under a microscope (40× magnification), and analyzed by flow cytometry (FACS CANTO BD) for T CD8+ lymphocyte expression after 52 days of infection. Results shown as percentage and total population of cells positive for these markers in CD3+ marker-positive lymphocytes. (A) Percentage of CD8+ T lymphocytes; (B) Total CD8+ T lymphocyte population; Values show the mean ± standard deviation for each group. * P < 0.05, ** P < 0.01 indicate a significant difference between the groups in relation to the control (PBS); # P < 0.05, ## P < 0.01, ### P < 0.005 indicate a significant difference between the groups in relation to AD-MSC; + P < 0.05 indicates a significant difference in the AD-MSC group in relation to the PA group. [file 13287_2020_1889_MOESM2_ESM.pdf]

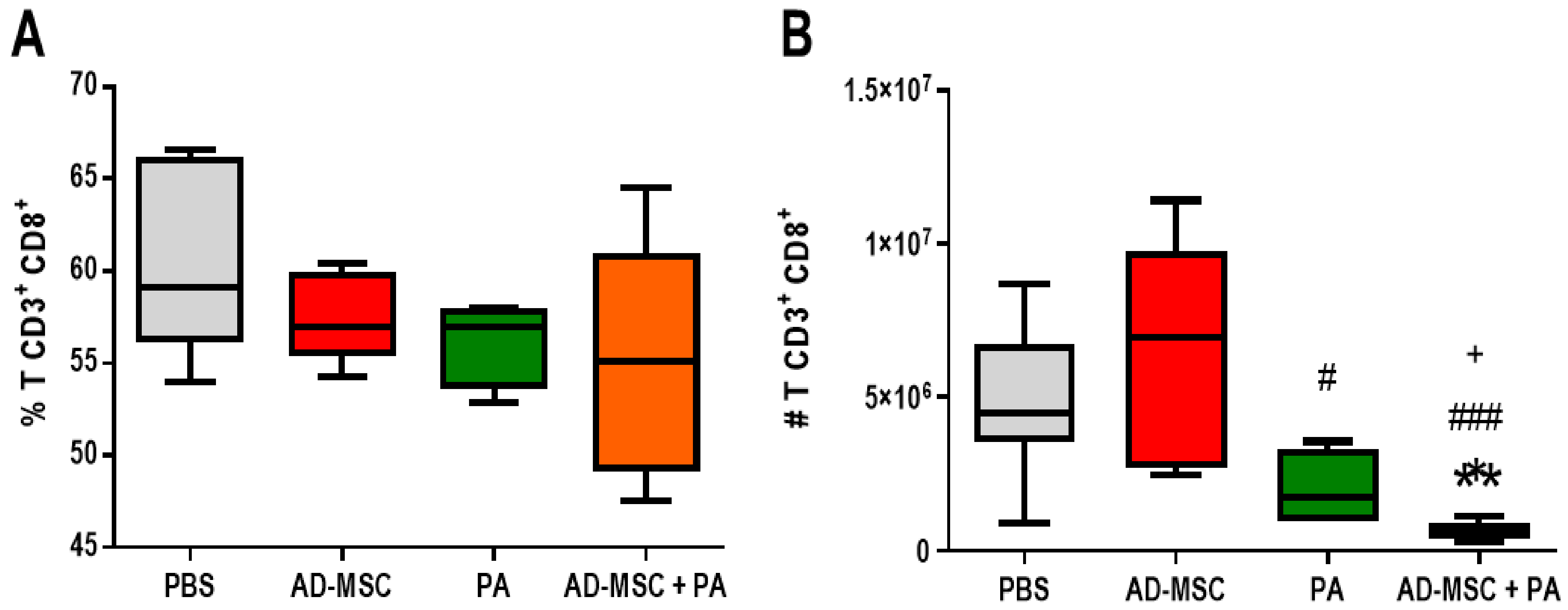

Additional File 2: Figure S2: Detection of T CD4$^+$ and T$_{reg}$ lymphocytes by flow cytometry (FACS). Cells were collected from the draining popliteal lymph node macerate, counted under a microscope (40× magnification), and analyzed by flow cytometry (FACS CANTO BD) for T CD8$^+$ lymphocyte expression after 52 days of infection. Results shown as percentage and total population of cells positive for these markers in CD3$^+$ marker-positive lymphocytes. (A) Percentage of CD8$^+$ T lymphocytes; (B) Total CD8$^+$ T lymphocyte population; Values show the mean ± standard deviation for each group. * P <0.05, ** P <0.01 indicate a significant difference between the groups in relation to the control (PBS); # P <0.05, ## P <0.01, ### P <0.005 indicate a significant difference between the groups in relation to AD-MSC; + P <0.05 indicates a significant difference in the AD-MSC group in relation to the PA group.
